# Effects of Lipidation on a Proline-Rich Antibacterial Peptide

**DOI:** 10.3390/ijms22157959

**Published:** 2021-07-26

**Authors:** Federica Armas, Adriana Di Stasi, Mario Mardirossian, Antonello A. Romani, Monica Benincasa, Marco Scocchi

**Affiliations:** 1 Department of Life Sciences, University of Trieste, 34127 Trieste, Italy; armas@smart.mit.edu (F.A.); ADRIANA.DISTASI@phd.units.it (A.D.S.); mmardirossian@units.it (M.M.); mbenincasa@units.it (M.B.); 2 Area Science Park, Padriciano, 34149 Trieste, Italy; 3 Antimicrobial Resistance Interdisciplinary Research Group, Singapore-MIT Alliance for Research and Technology, Singapore 138602, Singapore; 4 Department of Medical Sciences, University of Trieste, 34129 Trieste, Italy; 5 Arta Peptidion srls, 43126 Parma, Italy; artapeptidion@gmail.com

**Keywords:** lipidation, antimicrobial peptide, lipopeptide, membrane permeabilization, antibiotic resistance, antimicrobial agent, broad-spectrum activity

## Abstract

The emergence of multidrug-resistant bacteria is a worldwide health problem. Antimicrobial peptides have been recognized as potential alternatives to conventional antibiotics, but still require optimization. The proline-rich antimicrobial peptide Bac7(1-16) is active against only a limited number of Gram-negative bacteria. It kills bacteria by inhibiting protein synthesis after its internalization, which is mainly supported by the bacterial transporter SbmA. In this study, we tested two different lipidated forms of Bac7(1-16) with the aim of extending its activity against those bacterial species that lack SbmA. We linked a C12-alkyl chain or an ultrashort cationic lipopeptide Lp-I to the C-terminus of Bac7(1-16). Both the lipidated Bac-C12 and Bac-Lp-I forms acquired activity at low micromolar MIC values against several Gram-positive and Gram-negative bacteria. Moreover, unlike Bac7(1-16), Bac-C12, and Bac-Lp-I did not select resistant mutants in *E. coli* after 14 times of exposure to sub-MIC concentrations of the respective peptide. We demonstrated that the extended spectrum of activity and absence of de novo resistance are likely related to the acquired capability of the peptides to permeabilize cell membranes. These results indicate that C-terminal lipidation of a short proline-rich peptide profoundly alters its function and mode of action and provides useful insights into the design of novel broad-spectrum antibacterial agents.

## 1. Introduction

The consequences of the spread of multidrug-resistant pathogens are recognized as a global health problem and reduce the chances of successful treatment of infections [1]. Moreover, resistance to last-resort antibiotics, such as colistin, for the treatment of Gram-negative infections is spreading rapidly [2,3]. In this scenario, it is crucial to develop molecules with new or improved activities against bacteria and low toxicity against the host. Antimicrobial peptides (AMPs), also known as host defence peptides (HDPs), are natural defence effectors produced by animals and plants to counteract microorganisms [4,5] and have been proposed as potential alternatives to conventional drugs [6,7].

Most AMPs are cationic and amphipathic molecules that are attracted to the negatively charged bacterial membranes, resulting in perturbation and damage to the lipid bilayer [8]. As a result, AMPs generally exhibit a broad spectrum of activity and low selectivity against cell targets [9]. In contrast, the group of proline-rich antimicrobial peptides (PrAMPs) act on bacterial intracellular targets without apparent membrane damage [10,11]. Among PrAMPs, those from insects and mammals represent the best studied examples [12,13,14].

Several PrAMPs have been shown to require specific bacterial membrane proteins (such as SbmA and the MdtM complex) to enter the bacterial cytoplasm [15,16,17], and this restricts their antibacterial activity to bacteria, among the Gram-negative, that express these transporters [18]. Within bacterial cells, PrAMPs inhibit the elongation phase of protein synthesis by binding in the exit tunnel of the ribosome [19,20,21,22] and probably other functions associated with the chaperone DnaK [23].

Some N-fragments of the mammalian PrAMP Bac7, Bac7(1-16) and Bac7(1-35), display antimicrobial activity at micromolar concentrations against Gram-negative pathogens including *Acinetobacter baumannii*, *Salmonella* spp., and *Klebsiella pneumoniae*, but not against *Staphylococcus aureus* and other Gram-positive species [24,25,26,27].

Narrow-spectrum antibacterial agents may attenuate the selection and spread of bacterial resistance if the causative agent of the infection is known. However, in severe infections where treatment must be initiated before the pathogen is identified, a broad-spectrum agent is preferable [28,29]; hence the need to optimize PrAMPs to broaden their spectrum of activity. The natural antimicrobial efficacy and safety of AMPs have been improved by various strategies [30]. A well-documented method to enhance the efficacy of AMPs is the conjugation of the peptide with aliphatic fatty acids, resulting in synthetic lipopeptides [31]. Studies of synthetic lipopeptides evidenced that acylated AMPs exhibit improved cell selectivity and excellent activity, especially against Gram-positive bacteria [32,33,34].

Previously, we screened a library of Arg-rich ultrashort cationic antimicrobial lipopeptides (USCLs) based on the Arg-X-Trp-Arg-NH_2_ peptide moiety conjugated to a fatty acid. Lp-I was identified as the candidate that exhibited the highest antimicrobial activity against Gram-positive bacteria with low cytotoxicity [35]. In contrast, adding a lipid moiety to PrAMPs to broaden their spectrum of activity has hardly been investigated. A first attempt was made by linking aliphatic groups to the N-terminus of short consensus sequences derived from different PrAMPs, resulting in new derivatives with moderate antimicrobial activity [36].

In this study, we investigated the effects of lipidation of the PrAMP Bac7(1-16) to extend its antimicrobial activity to bacterial species lacking dedicated transport systems. Two different derivatives were synthesized, in which Bac7(1-16) was linked at its C-terminus to a C12-alkyl chain or to the ultrashort cationic lipopeptide Lp-I. The lipidated forms were tested for their antimicrobial activity against a broad panel of bacterial species, their cytotoxicity was assessed on human erythrocytes and nucleated cells, and their tendency to select bacterial resistance was evaluated. The results of this study indicated that the addition of a lipid component to the peptide strongly influences not only the spectrum of activity, but also the mechanism of action and the rate of appearance of resistant mutants. These findings contribute to the understanding of the effects of PrAMP lipidation and will be useful in the development of innovative and more effective antimicrobial agents.

## 2. Results

### 2.1. Bac-C12 and Bac-Lp-I Design

**Figure 1:**
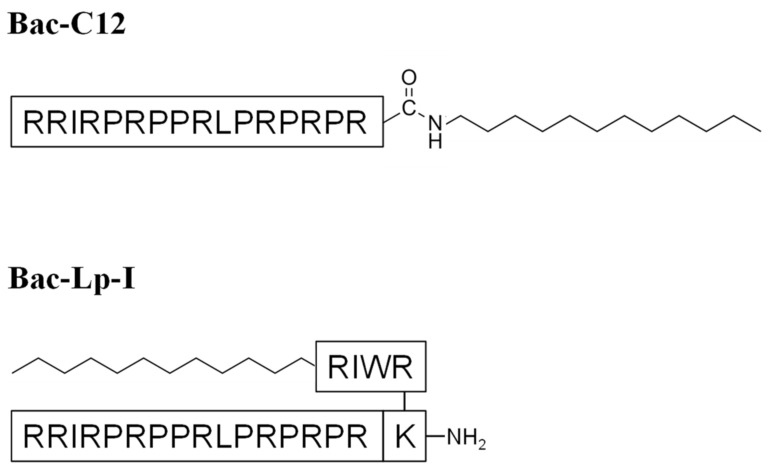
Chemical structures of Bac-C12 (MW: 2244.03) and Bac-Lp-I (MW: 2998.02).

Two different lipidated Bac7(1-16) derivatives were designed as schematically shown in Figure 1. The C-terminus of Bac7(1-16) was linked by an amide bond to a dodecylamine (C12-alkyl chain), obtaining the lipopeptide Bac-C12. The length of the hydrocarbon tail (C12) allowed to obtain sufficient solubility of the molecule. The second derivative, Bac-Lp-I, was designed by joining Bac7(1-16) and the previously characterized ultrashort lipopeptide Lp-I [35], constituted by the tetrapeptide RWIR linked at the N-terminus to a C12 fatty acid. Lp-I was linked to Nε of Bac7(1-16) modified at the C-terminus with an additional C-terminal lysine (Figure 1).

### 2.2. Antimicrobial Activity of Bac-C12 and Bac-Lp-I

We compared the antimicrobial activity of the two different lipidated forms of Bac7(1-16) with that of the original peptides Bac7(1-16) and Lp-I against a panel of Gram-positive and Gram-negative reference strains and against a clinical isolate of *Escherichia coli* O18K1H7 (Table 1). In general, both Bac-C12 and Bac-Lp-I displayed a broadened spectrum of activity against Gram-positive and Gram-negative bacteria and, in most cases, better antimicrobial activity than the original molecules.

Bac-C12 and Bac-Lp-I were more active than the single native peptides against *Staphylococcus epidermidis* ATCC 12228, *Burkholderia cepacia* J2315, *E. coli* O18K1H7, *Pseudomonas aeruginosa*, and *Acinetobacter baumannii* strains, with MIC values ranging from 2 μM to 8 μM. In contrast, lipidation did not increase efficacy against *Bacillus subtilis* DSMZ 4181, *E. coli* ATCC 25922, and *Salmonella enterica* serovar typhimurium ATCC 14028, where Bac7(1-16) MICs were equal to or twofold better than the lipidated variant.

Interestingly, lipidation strongly increased activity against Gram-positive *Enterococcus faecalis* ATCC 29212, *Staphylococcus aureus* strains and *S. epidermidis* ATCC 12228 (MIC range 2–8 μM), which were resistant to Bac7(1-16) (MIC 64 μM). Bac-C12 showed higher antimicrobial activity than Bac-Lp-I against *S. aureus* ATCC 29213, *Stenotrophomonas maltophilia* and *B. cepacia*. No major differences were observed in other cases, suggesting that the presence of the C-12 lipid tail played a more important role in improving antimicrobial activity than the addition of the lipotetrapeptide RWIR (Lp-I) (Table 1).

Overall, these results indicate an impressive enlargement of the inhibitory activity of the two lipidated forms of Bac7(1-16), extending to bacterial species lacking the bacterial transport system [17]. The only exception was *B. subtilis*, although it is generally recognized as a nonpathogenic strain [37].

**Figure 2:**
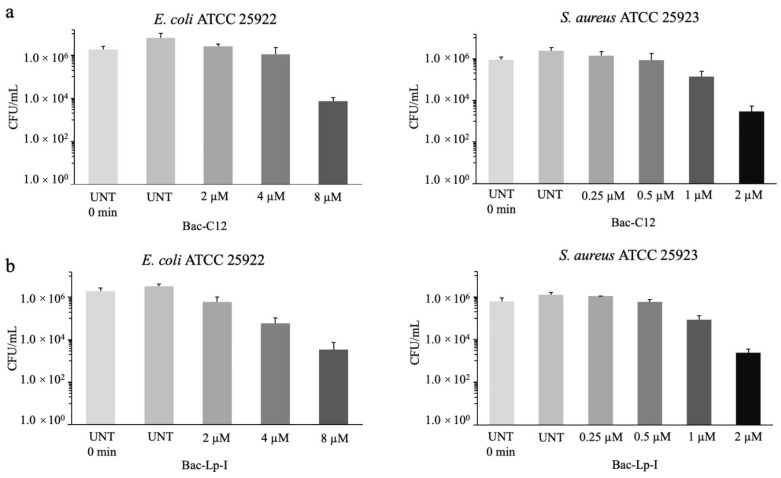
Evaluation of the bactericidal activity of Bac-C12 and Bac-Lp-I on *E. coli* ATCC 25922 and *S. aureus* ATCC 25923. The bactericidal activity with Bac-C12 (panel **a**) and Bac-Lp-I (panel **b**) on *E. coli* ATCC 25922 and *S. aureus* ATCC 25923 cells was determined using a mid-logarithmic phase bacterial suspension, diluted in fresh MHB to a final concentration of 10^6^ CFU/mL, and incubated for 30 min at 37 °C with increasing concentrations of peptide. Samples were then diluted in PBS, plated on MH agar, and incubated overnight to allow the colony counts. UNT = untreated cells. Data are the mean ± SD of three independent experiments.

Both Bac-C12 and Bac-Lp-I showed bactericidal values (MBC) similar to or twofold higher than MICs, suggesting bactericidal activity of these derivatives. The bactericidal activity of Bac-C12 and Bac-Lp-I was also determined against *E. coli* ATCC 25922 and *S. aureus* ATCC 25923 using the viable colony count method. A 30 min exposure to Bac-C12 caused a 2.9-log reduction in *E. coli* and *S. aureus* cells (CFU/mL) at 8 μM and 2 μM, respectively, values close to the MICs (Figure 2, panel a). The effect of Bac-Lp-I against the same strains was very similar to that of Bac-C12. This derivative started to reduce the cell number of *E. coli* and *S. aureus* at 4 μM and 1 μM, respectively, at concentrations close to the MIC (Figure 2, panel b). These results indicate that lipidation does not lower the bactericidal capability of Bac7(1-16) that has been previously determined [27].

### 2.3. Evaluation of Cytotoxicity of Bac-C12 and Bac-Lp-I

**Figure 3:**
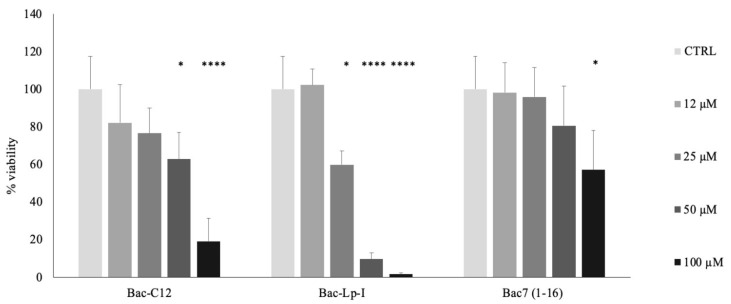
Cell viability of human epidermal keratinocytes HaCaT cells treated with Bac-C12, Bac-Lp-I, or Bac7(1-16). Cell viability was evaluated after 24 h incubation with indicated concentrations of peptides by using the MTT assay. PBS was used as a control. The cell viability is expressed as a percentage of MTT absorbance (570 nm) measured on untreated cells (ctrl) assumed as 100% viability. Each value represents the mean ± SEM of three independent experiments performed in triplicate. * *p* < 0.05 vs. untreated cells (ctrl), and **** *p* < 0.0001 vs. untreated cells (ctrl) (Kruskal–Wallis test and ANOVA).

We evaluated the possible cytotoxic effects of Bac-C12 and Bac-Lp-I on human red blood cells (hRBCs) and human keratinocytes. Both lipidated peptides displayed ≤3% hemolysis even at 100 μM, the highest concentration tested, indicating that Bac-C12 and Bac-Lp-I do not damage hRBCs even at concentrations far above the microbicidal ones (Figure S1, Supplementary Materials). We then assayed the effects of the peptides on human epidermal keratinocytes (HaCaT). HaCaT cells were exposed to Bac-C12, Bac-Lp-I, or Bac7(1-16) for 24 h at various concentrations. Bac-Lp-I reduced cell viability starting at 25 μM (decrease of approximately 40%) with a further decrease in viability at 50 and 100 μM. Bac-C12 was found to be non-toxic at 25 μM but reduced keratinocyte viability at 50 μM (decrease of approximately 40%) (Figure 3). Since Bac7(1-16) affected the viability of the same cells starting from 100 μM (Figure 3), these results suggest that the addition of a lipid tail has some effects on host cells and that lipopeptides are less selective for their cell target. The addition of C-12 alone to Bac7(1-16) had a less pronounced effect than the addition of lipotetrapeptide (Bac-Lp-I). In either case, both lipidated forms acted on cells at concentrations 3–8-fold higher than the microbicidal ones. The biocompatibility of Bac-C12 was also assayed against the human line of lymphocyte precursors MEC-1. Similar results were obtained, with the decrease in cell viability at 32 μM (Figure S2, Supplementary Materials) confirming the trend highlighted in Figure 3.

### 2.4. Resistance Selection in E. coli by Bac-C12 and Bac-Lp-I

Since bacteria become resistant to most antibiotics quite quickly, we have investigated whether Bac7(1-16) derivatives at sub-MIC concentrations could select for de novo resistance in bacteria within a few generations. To detect possible appearance of resistant mutants, bacterial cultures of *E. coli* ATCC 25922 were subjected to 14 consecutive MIC assays performed as described in the Materials and Methods section and the presence of resistant mutants was reported as an increase in relative MIC.

**Figure 4:**
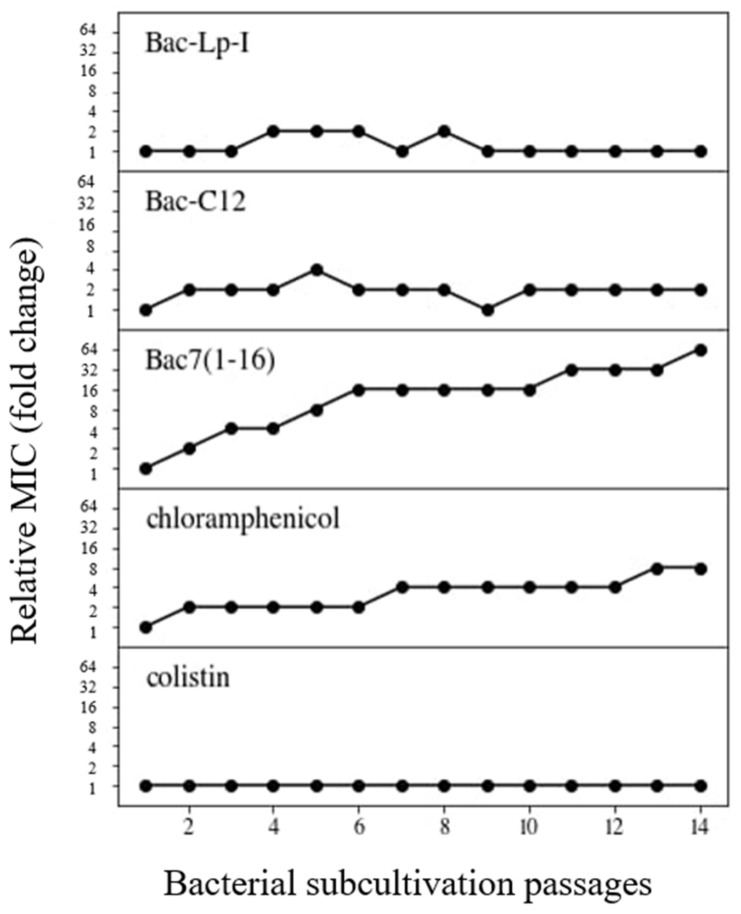
Susceptibility variation of *E. coli* ATCC 25922 to different lipopeptides and antibiotics. Relative MIC values represent the MIC fold-change observed during 14 passages of bacterial subcultures compared to the original MIC value against the wild-type. Bacterial cells grown at the highest concentration of the antimicrobial (1/2MIC) were subcultured and used for the next MIC assay. The procedure was repeated for 14 passages. Chloramphenicol and colistin were used as reference antibiotics for non-lytic and lytic mode of action, respectively. Each plot represents a representative result of assays repeated at least two times. Whole data are reported in Table S1, Supplementary Materials.

Within the *E. coli* passages, a 2-fold increase in MIC values was observed for Bac-C12, while MIC of Bac-Lp-I did not increase at all and, if anything, showed a 2-fold variation (Figure 4 and Table S1, Supplementary Materials). This behavior was similar to that of colistin, used as a reference for a membranolytic agent, where no increase in MIC values was observed. The relative MIC of Bac7(1-16) was 64-fold higher than the initial MIC, similarly to what was observed with chloramphenicol (Figure 4). In fact, the MIC of chloramphenicol increased from 4-fold to 64-fold within three independent experiments (Figure 4 and Table S1, Supplementary Materials).

Overall, these results indicate that bacteria become quite easily less susceptible to Bac7(1-16), while selection of a novel strain resistant to its derivatives Bac-C12 and Bac-Lp-I seems to be a more difficult event.

### 2.5. Assessment of the Integrity of the Bacterial Cell Membrane

**Figure 5:**
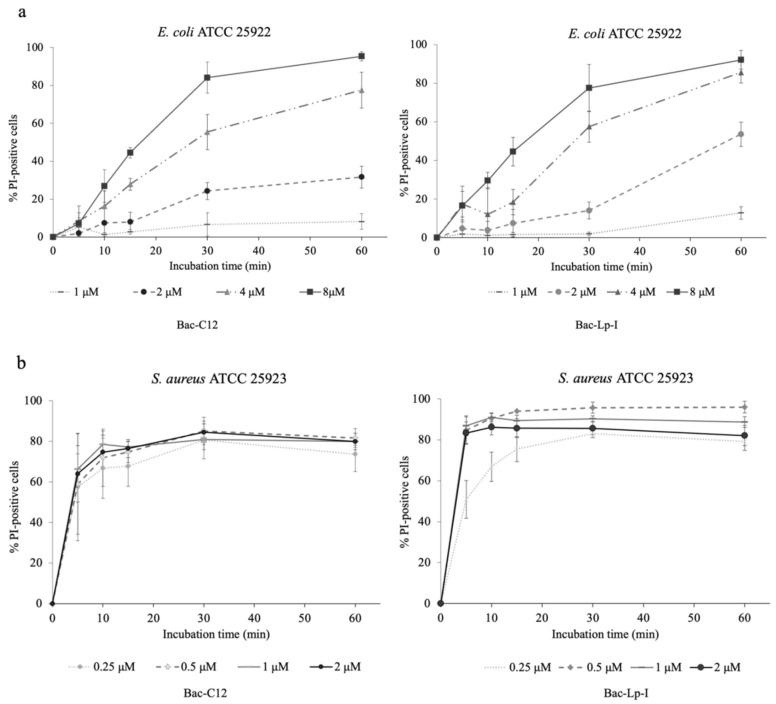
Membrane-damaging activity of Bac-C12 and Bac-Lp-I on *E. coli* ATCC 25922 (**a**) and *S. aureus* ATCC 25923 (**b**). The permeabilization assay with Bac-C12 and Bac-Lp-I has been performed in MHB. Bacterial cells were treated, for different incubation times, with indicated concentrations of peptide. % PI-positive: percentage of propidium iodide-positive cells. The background level of permeabilized cells, obtained with untreated samples, was always below 2% and was subtracted to the corresponding peptide-treated sample. Data are the mean ± SD of four independent experiments.

Since the addition of an alkyl chain to peptides is expected to increase their interaction with membranes, we examined the effects of lipopeptides on membrane integrity. The propidium iodide uptake assay was performed on *E. coli* ATCC 25922 and *S. aureus* ATCC 25923 used as representatives of Gram-negative and Gram-positive strains, respectively, and exposed to Bac-C12 or Bac-Lp-I at the same concentrations used in killing experiments. Bac-C12 and Bac-Lp-I increased the number of PI-positive cells within a few minutes and in a concentration-dependent manner (Figure 5), indicating that both lipopeptides acquired the capability to permeabilize bacterial cells at the same or even lower bactericidal concentrations. By contrast, the original Bac7(1-16) did not increase the number of permeabilized cells (data not shown), in agreement with previous data [27].

Very similar effects on membranes were observed between Bac-C12 and Bac-Lp-I. Both peptides permeabilized more than 80% of *E. coli* cells after 30 min of treatment at MIC concentration (4–8 μM) (Figure 5, panel a). An even higher degree of permeabilization was observed in *S. aureus* cells. Bac-C12 and Bac-Lp-I damaged the cells already at 0.25 μM, a sub-MIC concentration. Bac-Lp-I at 0.5 μM permeabilized 80% of the cells within only a 10 min incubation, while Bac-C12 at 0.5 μM achieved the same percentage of permeabilized cells within 20 min (Figure 5, panel b). These results clearly show that the addition of the lipid chain dramatically changes the behavior of both derivatives and clearly suggest that cell permeabilization has become the main mechanism of killing.

## 3. Discussion

PrAMPs are in the spotlight for the development of new antibiotics, but their narrow spectrum of activity may hinder their further progress toward clinical use [14,18]. Therefore, in recent years, great efforts have been made to expand the antimicrobial spectrum of PrAMPs by introducing sequence modification, residue substitution, or both [27,38,39,40,41]. In this study, we tested the effect of lipidation on the mammalian PrAMP Bac7(1-16) and demonstrated that the addition of a lipid chain to Bac7(1-16) greatly enhanced its antibacterial activity. We also demonstrated that the improvement was likely associated with a modification of the killing mechanism.

Both the C12-alkylated Bac7(1-16) and the chimeric Bac-Lp-I lipopeptide displayed higher antimicrobial activity against some pathogenic species and a broad spectrum of activity targeting a larger number of bacterial species, including Gram-positive bacteria. The molecules showed an MIC of ≤ 8 μM against all bacterial species tested, regardless of whether they were Gram-positive or Gram-negative (see Table 1). In addition, the panel of pathogens tested included among the reference strains also most of the ESKAPE listed by WHO as species of greatest concern for human health [42]. Thus, Bac-C12 and Bac-Lp-I was shown to inhibit pathogens of relevant clinical interest. The extended spectrum of activity is probably related to the capability of Bac-C12 and Bac-Lp-I to act independently of the presence of the bacterial membrane proteins SbmA or MdtM. Thus, the antimicrobial mechanism of these lipopeptides acting directly on bacterial membranes is not limited to the few bacterial species expressing these transport proteins. This is also an interesting result because any mutation of the transporter that affects peptide internalization could lead to decreased susceptibility to these PrAMPs [43]. A highly efficient system of peptide transport through SbmA for the non-lytic native Bac7(1-16) might also explain the reason why the *E. coli* strains 25922 and BW25113 continued to be more susceptible to Bac7(1-16) than to its lipidated derivatives. Further studies, such as the determination of the level of expression of SbmA in the distinct *E. coli* stains, could be useful to clarify this point as well as the high potency of Bac7(1-16) against *B. subtilis* DSMZ 4181.

The lipidated derivatives were designed using the previously characterized PrAMP Bac7(-1-16) [24,27]. The C12 lipid tail was added to the C-terminus as it is known to play a minor role in ribosome inhibition and antimicrobial activity of the peptide [22]. The second lipopeptide, synthesized by linking Bac7(1-16) with the ultrashort lipopeptide Lp-I [35], was designed with the aim of obtaining a new molecule that had the sum of the activities of the individual peptides. The results showed that there is a slight difference between the antibacterial activity of Bac-C12 and Bac-Lp-I, suggesting that the addition of the alkyl-C12 chain rather than the addition of the ultrashort lipopeptide plays the prevalent role in enhancing the antimicrobial activity (see Table 1).

Lipidation is a commonly used approach to enhance or modulate the antimicrobial activity of AMPs such as the α-helical peptides (comprehensive review in [33]). In contrast, there is limited information on PrAMP modified by lipids. Short proline-rich lipopeptides were developed by Domalaon et al. by combining a PR-rich heptapeptide with N-terminal fatty acids of different lengths [36]. Of the lipopeptides tested, only those with a C-18 chain exerted moderate antimicrobial activity, while those with a C-12 chain were not active except when tested in combination with other compounds [36]. These results are consistent with previous observations highlighting the importance of a free N-terminus in the antimicrobial activity and mechanism of action of mammalian PrAMPs [21,44,45]. Moreover, the weak activity of the lipidated PR-rich heptapeptide suggests that a seven-residue peptide is too short and reinforces the observation that 16–17-residue peptides are the shortest ones that exhibit antimicrobial activity [21,24,44].

Mode of action analysis showed that lipidation of Bac7(1-16) clearly transformed a non-permeabilizing peptide into a markedly membrane-active molecule. Both lipidated forms destabilized the bacterial membranes of *E. coli* and, more pronouncedly, those of *S. aureus* even at sub-MIC concentrations, strongly indicating that membrane permeabilization is a process clearly correlated with bacterial killing. Interestingly, the kinetics of bacterial membrane permeabilization by Bac-C12 were very similar to those observed in the same microorganisms using the lipopeptide Lp-I [35], suggesting that the two molecules have a very similar mechanism of action.

The other two effects of Bac-C12 and Bac-Lp-I noted in this study, i.e., the lack of appearance of resistant mutants within 14 consecutive passages and the increased cytotoxicity, are much likely related to the mechanism of membrane permeabilization.

Indeed, both lipopeptides did not allow selection of de novo resistance in the treated *E. coli* cultures even after long exposure (14 passages), which is thus an interesting and desirable property for antibiotics. This behavior is very similar to that of the well-known permeabilizing antibiotic colistin. In contrast, Bac7(1-16), which has specific targets for internalization and activity, rapidly increased its MIC value under the same experimental conditions. This result is consistent with a modification of the killing mechanism by the addition of the lipid moiety and the acquisition of non-specific targets such as the bacterial membrane. Resistance to many AMPs can evolve at measurable rates, at least in vitro [46]. However, the MIC increase in mutants for membrane-acting AMPs is lower than that for antibiotics with specific molecular targets. Our results suggest that the acquisition of a lipid chain reduces the rates of mutant selection. This observation is consistent with a study performed on the longer fragment Bac7(1-35) [26]. This peptide is less dependent than Bac7(1-16) on SbmA for its internalization [17] and at high concentrations also exerts membrane destabilizing activity [47]. It has been shown that also Bac7(1-35) did not select resistance in *A. baumannii* AB5075 strain after 14 passages.

The addition of the two lipid moieties also affects the biocompatibility of Bac7(1-16) toward host cells. Bac-C12 and, more significantly, Bac-Lp-I began to reduce cell viability of keratinocytes at 2–4 lower concentrations than the peptide alone. This result was quite expected, since the introduction of an alkyl chain into antimicrobial peptides often enhances the cytotoxic effects [48]. On the other hand, the difference in cytotoxicity between Bac-C12 and Bac-Lp-I was not obvious. It was generally observed that the cytotoxicity of lipopeptides increases with the length of their hydrophobic chain [48]. The tryptophan residue present in Lp-I and the linker used to link Bac7(1-16) and Lp-I contribute to increase the hydrophobicity of the molecule, perhaps making it more cytotoxic compared to Bac-C12.

It remains unclear what properties the peptide moiety in lipopeptides should have to optimize their activity. To date, it has not been possible to draw general lines and predict the behavior of antimicrobial lipopeptides, as these molecules can show very different effects due to the abundance of factors to be considered [48]. For example, in the work of Grimsey and colleagues, the same C-terminal lipidation process with a C-12 chain that improved the antimicrobial efficacy of one peptide was detrimental to another [49]. Although our data suggest that the addition of lipid moieties to Bac7(1-16) promotes a lytic mode of action, the role of the proline-rich peptide moiety in lipopeptides remains uncertain. The effect of PrAMP may be limited to the fact that it is a generic cationic peptide moiety that has sufficient length and a good ratio between polar and apolar residues and that, in combination with the proper lipid chain, exerts its antimicrobial action by destabilizing the bacterial envelope, as many other lipopeptides do. This idea can be supported by some similarities between the lipopeptides discussed in this work and other lipopeptides with quite comparable peptide and lipid contents. The MIC values exhibited by lipopeptide (RW)3K-C12 against reference strains of *S. aureus*, *E. coli*, *P. aeruginosa*, and *A. baumannii* are very similar to those of the lipopeptides synthesized in the current work [50]. The same amino acid sequence bearing the lipidated Lys at the N-terminus also showed similar activity [50]. The synthesized lipopeptide binding a C12 to the N-terminus of the peptide NH_2_-KRRVRWIIW-CONH_2_, had a profile of antimicrobial activity and toxicity quite comparable to that of Bac-C12 and Bac-Lp-I. Indeed, lipidation moderately improved the antimicrobial effect of NH_2_-KRRVRWIIW-CONH_2_ toward *S. aureus*, *E. faecalis*, *P. aeruginosa*, and *S. typhimurium*, whereas it slightly worsened activity toward *E. coli*. On the other hand, the fatty chain made the lipopeptide more cytotoxic than the native molecule [49].

A comparison of pros and cons between Bac-C12 and Bac-Lp-I revealed that Bac-C12 is the most promising compound for future development. Bac-C12 has an interestingly broad spectrum of activity and potent antibiotic activity and displayed only a modest decrease in biocompatibility compared to Bac7(1-16). Moreover, the synthesis of Bac-C12 is simpler than that of Bac-Lp-I. It is performed in a single step and requires the formation of only one amide bond, unlike Bac-Lp-I, which requires the linkage of two more complex compounds. Overall, these properties make it appealing not only on its own but also for possible use in combination with non-lipidated PrAMPs.

In conclusion, this study indicates that lipidation of PrAMPs is a useful strategy to enhance PrAMP activity by deeply modifying the mechanism of action and providing interesting antimicrobial molecules. Therefore, Bac-C12 deserves further studies to explore its potential as a lead compound that can be used in the new generation of antibiotic therapy.

## 4. Materials and Methods

### 4.1. Peptides Synthesis

Bac-C12, Bac-Lp-I, and Bac7(1-16) (95% pure) were purchased from NovoPro Bioscience (Shanghai, China). The purity level was verified by RP-HPLC and ESI-MS analyses. Lp-I was purchased from GenScript (Piscataway, NJ, USA) and purified to 90% purity by RP-HPLC [35]. Lipopeptides and Lp-I were dissolved in dimethyl sulfoxide (DMSO) following the manufacturer’s instructions. Any effects of DMSO on bacterial growth were excluded by control assays performed using the same DMSO concentrations present in the peptide dilutions. Bac7(1-16) was resuspended in sterile milliQ water. The chemical structures for Bac-C12 and Bac-Lp-I are shown in Figure 1.

### 4.2. Bacterial Culture

The bacteria used in this study, which were purchased from American Type Culture Collection (Manassas, Virginia, USA), the German Collection of Microorganisms and Cell Cultures (Leibniz Institute, Braunschweig, Germany), or Keio Collection, Japan [51], were as follows: *Bacillus subtilis* DSMZ 4181, *Enterococcus faecalis* ATCC 29212, *Staphylococcus aureus* ATCC 25923 and 29213, *Staphylococcus epidermidis* ATCC 12228, *Acinetobacter baumannii* ATCC 17978 and 19606, *Burkholderia cenocepacia* J2315, *Escherichia coli* ATCC 25922, BW25113, BW25113 Δ*sbmA* [51], and O18K1H7, *Klebsiella pneumoniae* ATCC 13883 and 700603, *Pseudomonas aeruginosa* ATCC 27853 and PAO1, *Salmonella typhimurium* ATCC 14028, and *Stenotrophomonas maltophilia* ATCC 13637. Overnight cultures of each strain were diluted 1:30 in fresh Mueller–Hinton broth (MHB; Difco) and incubated at 37 °C with shaking (140 rpm) for approximately 2 h (mid-log phase) until an optical density (OD) of ≈ 0.3 at 600 nm was achieved. *E. coli* BW25113 *ΔsbmA* was grown in MHB with the addition of 50 μg/mL kanamycin (Sigma, Milan, Italy).

### 4.3. Evaluation of the Antimicrobial Activity

Minimum inhibitory concentration (MIC) assays were performed as previously reported [24]. Each peptide was separately dispensed in the first wells of a 96-well microtiter plate (Sarstedt, Milan, Italy) and serially diluted twofold in MHB into the subsequent wells. A mid-log phase bacterial suspension (2.5 × 10^5^ CFU/mL) was added in MHB to the microtiter plate wells and the microtiter plates were incubated at 37 °C. The MIC value was taken as the lowest peptide concentration that inhibited visible growth after 18 h. MIC values for *B. cepacia* and *S. maltophilia* were determined after 48 h incubation. Data represent the mode of at least three independent experiments.

The minimal bactericidal concentration (MBC) assay was performed after MIC. 10 μL aliquots from the wells without visible bacterial growth were seeded in Mueller–Hinton agar (MH agar; Difco) and incubated for 24 h to allow colony growth. The peptide concentration causing a reduction ≥ 99.9% of the initial inoculum bacteria was defined as MBC value (CLSI M26-A, Methods for Determining Bactericidal Activity of Antimicrobial Agents) as previously described [52]. Data are reported as the mode of at least three independent experiments. The bactericidal activity of the peptides was determined using a bacterial suspension in the mid-log phase of *S. aureus* ATCC 25923 or *E. coli* ATCC 25922 cells diluted to 1 × 10^6^ CFU/mL in fresh MHB and incubated at 37 °C with each peptide used at concentrations corresponding to their MIC or sub-MIC values. After 30 min incubation, samples were diluted in phosphate-buffered saline (PBS), plated on MH agar, and incubated overnight to determine colony counts. Data represent the mean ± SD of three independent experiments.

### 4.4. In Vitro Toxicity Assays

Hemolysis assays were performed using human red blood cells (hRBCs) following the protocol of Laverty [31]. Briefly, hRBCs from human whole blood from a single donor (Cambridge Bioscience, UK) were pelleted, washed three times with equal volumes of PBS by centrifugation (15 min, 900× *g*), and then suspended at 8% (*v/v*) in PBS. 100 μL aliquots of the hRBC-PBS suspensions were added to each well of the 96 microtiter plates (Euroclone, Milan, Italy), followed by 100 μL of the lipopeptide in PBS at different concentrations. Samples were incubated at 37 °C for 1 h, then centrifuged at 1000× *g* (Sigma 4K15C) for 10 min, and 100 μL aliquots of the supernatant were transferred to a new 96-well microtiter plate. Hemoglobin release was measured as absorbance values at 540 nm using a Tecan microplate reader (Tecan Trading AG, Switzerland). 0% and 100% hemolysis were determined using PBS and a 1% solution of Triton X-100, respectively. Results are reported as mean ± SD of two independent experiments performed in triplicate.

Cytotoxicity was evaluated by the tetrazolium salts test (MTT) (Sigma) assay. Human immortalized epidermal keratinocyte HaCaT cells (DKFZ, Eppelheim, Germany) and the human line of lymphocyte precursors MEC-1 (ACC -497; DSMZ, Germany) were grown in high-glucose Dulbecco’s modified Eagle’s medium (DMEM) (Euroclone, Milan, Italy) and RPMI (Sigma, Milan, Italy), respectively. Both media were supplemented with 10% fetal bovine serum (FBS; Sigma, Milan, Italy), 2 mM glutamine (Sigma, Milan, Italy), 100 U/mL penicillin (Sigma, Milan, Italy), and 100 μg/mL streptomycin (Sigma, Milan, Italy). The cells were maintained at 37 °C in the presence of 5% CO_2_ and in a humidified incubator. The cells were seeded in 96 flat-bottom microtiter plates (Euroclon, Milan, Italy e) at a density of 2 × 10^4^ cells/well. After 24 h, the cells were exposed to different concentrations of peptides in a complete medium and incubated for 24 h. For the last 4 h of incubation, 20 μL MTT solution (1 mg/mL in PBS; Sigma, Milan, Italy) was added to each well. After incubation, the plates were centrifuged (Sigma 4K15C) at 480× *g* for 10 min, the supernatant of each well was carefully removed, and 120 μL PBS was added to each well. The insoluble MTT crystals were solubilized using 100 μL 10% IGEPAL (Sigma, Milan, Italy) in 0.01N HCl, and the plates were incubated overnight at 37 °C. Absorbance values were measured at 570 nm using the plate reader spectrophotometer Nanoquant infinite M200pro (Tecan, Männedorf, Switzerland). Data are absorbance values expressed as percentages of the untreated controls and are the mean ± SD of three independent experiments with internal triplicates.

### 4.5. Membrane Integrity Analysis

Bacterial cell membrane integrity assessment was evaluated by measuring propidium iodide (PI) (Sigma, Milan, Italy) uptake using flow cytometry [47]. Briefly, bacterial cultures in the mid-log phase were diluted in MHB to 1 × 10^6^ CFU/mL, and were incubated at 37 °C with MIC and sub-MIC concentrations of the peptide for different time points. PI was added to all samples at a final concentration of 10 μg/mL. At the end of each time point incubation, the bacterial cells were analyzed using a Cytomics FC 500 instrument (Beckman-Coulter, Inc., Fullerton, CA, USA). Data analysis was performed using FCS Express3 software (De Novo Software, Los Angeles, CA, USA). Data are expressed as mean ± SEM.

### 4.6. Resistance Selection by Serial Passages

Susceptibility to the compounds was assessed daily, by performing a serial passage protocol as previously described [26]. Briefly, MIC values for wild-type *E. coli* ATCC 25922 were first determined and recorded. Bacterial cells growing in the presence of the highest concentration of the antimicrobial (1/2 MIC) were harvested and inoculated into fresh MHB. These bacteria were grown and subjected to another MIC assay incubating them for 18 h at 37 °C with each of the antimicrobials. After incubation, bacteria grown at 1/2 MIC were again subcultured and used in a new MIC assay. The process was repeated for 14 passages (days). The range of peptide concentrations used for the MIC assay was adjusted and increased according to the rising MIC values (if any). Colistin (Sigma, Milan, Italy) and chloramphenicol (Sigma, Milan, Italy) were used as drug references.

### 4.7. Statistical Analyses

Significance of differences among groups was assessed using Prism program (GraphPad Software, San Diego, CA, USA) and an analysis of variance between groups (Kruskal–Wallis test or Bonferroni, ANOVA). Values of *p* ≤ 0.05 were considered statistically significant.

## Figures and Tables

**Table 1:** Minimum inhibitory concentration (MIC) and minimal bactericidal concentration (MBC) of lipidated Bac7(1-16) derivatives and their parental peptides.

Bacteria Strain	MIC (μM)				MBC (μM)	
	Bac7(1-16)	Lp-I	Bac-C12	Bac-Lp-I	Bac-C12	Bac-Lp-I
*B. subtilis* DSMZ 4181	1	4	4	2	4	2
*E. faecalis* ATCC 29212	>64	8	8	4	8	8
*S. aureus* ATCC 25923	>64	4	4	2	8	4
*S. aureus* ATCC 29213	>64	4	2	4	4	4
*S. epidermidis* ATCC 12228	>64	8	2	2	2–4	2
*A. baumannii* ATCC 17978	16	32	8	2	16	4
*A. baumannii* ATCC 19606	64	32	8	4	8	4
*B. cepacia* J2315	>64	16	2	8	n.d.	8
*E. coli* ATCC 25922	2	8	8	4	8	8
*E. coli* BW25113	4	8	4	4	4	16
*E. coli* BW25113Δ*sbmA*	32	n.d.	4	4	4	4
*E. coli* O18K1H7 ^#^	>64	16	8	8	8	16
*K. pneumoniae* ATCC 700603	8	64	8	4	8	4
*K. pneumoniae* ATCC 13883	4	>64	4	4	32	8
*P. aeruginosa* ATCC 27853	>64	16	8	8	16	16
*P. aeruginosa* PAO1	>64	16	8	8	>64	16
*S. typhimurium* ATCC 14028	2	32	4	2	8–16	4
*S. maltophilia* ATCC 13637	>64	8	2	8	4	16

^#^ Clinical isolates. Results derived from at least three independent experiments carried out at least in duplicate. MIC and MBC were recorded after 18 h and 24 h of incubation, respectively, for all strains with the exception of *B. cepacia* and *S. maltophilia*, needing 48 h. MIC and MBC values are reported as mode of the results. n.d. = value not determined.

## Data Availability

Data is contained within the article or Supplementary Materials.

## Supplementary Materials

The following are available online at https://www.mdpi.com/article/10.3390/ijms22157959/s1.

## Abbreviations

AMPs	Antimicrobial peptides
CFU	Colony-forming unit
CLSI	Clinical & Laboratory Standards Institute
DMEM	Dulbecco’s modified Eagle’s medium
DMSO	Dimethyl sulfoxide
FBS	Fetal bovine serum
hRBCs	Human red blood cells
MBC	Minimal bactericidal concentration
MEC-1	Human chronic lymphocytic leukemic B line
MHB	Mueller–Hinton broth
MIC	Minimum inhibitory concentration
MTT	Tetrazolium salts test
PBS	Phosphate-buffered saline
PI	Propidium iodide
PrAMPs	Proline-rich antimicrobial peptides
USCLs	Ultrashort cationic lipopeptides
