# Supplementary material for: Effects of Lipidation on a Proline-Rich Antibacterial Peptide
Source: Int J Mol Sci. 2021 Jul 26;22(15):7959. doi: 10.3390/ijms22157959 (PMC8347091; doi:10.3390/ijms22157959)
Supplement: Supplementary file 1 [file ijms-22-07959-s001.zip › ijms-1297105-supplementary.pdf]

Supplementary Materials

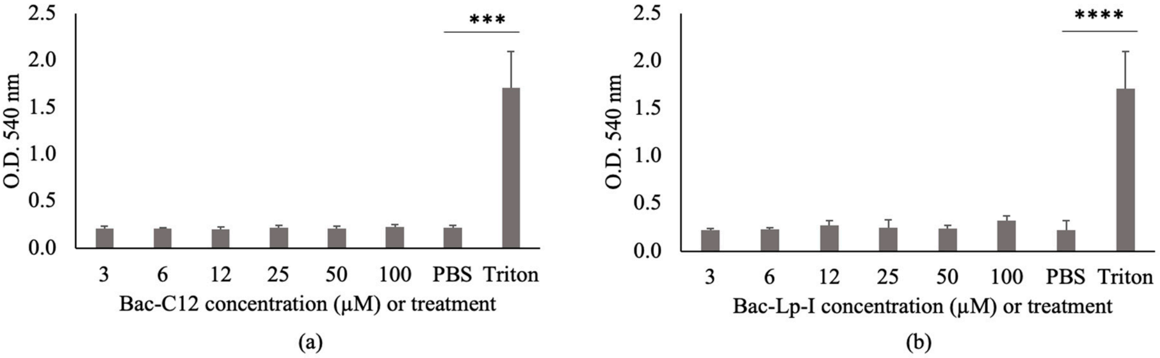

Figure S1. Haemolytic effect of Bac-C12 and Bac-Lp-I in a human red blood cell (hRBC) suspension. Hemolytic effect of Bac-C12 (**a**) and Bac-Lp-I (**b**) in a hRBC suspension. HRBC suspension without peptide treatments was used as a negative control (PBS). 1% Triton was used to obtain the 100% of hRBC hemolysis. (**a**) Absorbance at 540 nm of hRBC treated with increasing concentration of Bac -C12 showed not a significant increase of hemolysis compared to the PBS treated ones. (**b**) O.D. values at 540 nm of hRBC treated with increasing concentration of Bac-Lp-I showed no significant increase of hemolysis compared to the PBS treated ones. Data are mean ± SD of three independent experiments. *** $p< 0.001$ vs the PBS control, **** $p< 0.0001$ vs the PBS control (Kruskal–Wallis test, ANOVA).

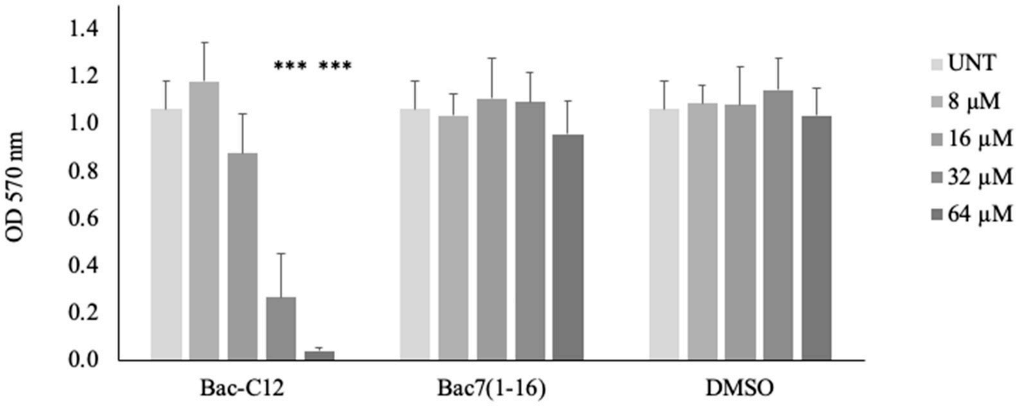

Figure S2. Cytotoxicity effects of Bac-C12 and Bac7(1-16) on the human chronic lymphocytic leukemic B line (MEC-1) MEC-1 cells. Cytotoxic activity was evaluated after 24h incubation with indicated concentrations of peptides. DMSO was a control and was used at the same percentages present in each peptide sample. The cell viability is expressed as OD measured at 570 nm. Each value represents the mean ± SD of 3 independent experiments performed in triplicate. *$p < 0.05$ vs untreated cells (UNT); **$p < 0.01$ vs untreated cells (UNT), ***$p < 0.001$ vs untreated cells (UNT), (Bonferroni, ANOVA).

Table S1. Susceptibility (MICs) of *E. coli* ATCC 25922 to different peptides and antibiotics after bacteria subcultures (passages 1-14). After each MIC assay, the bacteria present in the well corresponding to 1/2 MIC of the respective peptide or antibiotic were cultured and subjected to another MIC assay as described in *materials and methods* section. Chloramphenicol and colistin were used as reference antibiotics for non-lytic and lytic mode of action, respectively. Each row (with number I II and III) represents an independent experiment.

		MIC (µM)													
**n. passage →**		**1**	**2**	**3**	**4**	**5**	**6**	**7**	**8**	**9**	**10**	**11**	**12**	**13**	**14**
**Compound**	**n. test**														
**Bac-C12**	I	4	8	8	8	16	8	8	8	4	8	8	8	8	8
	II	2	4	4	4	4	4	8	2	4	4	4	4	8	4
**Bac-Lp-I**	I	4	4	8	8	4	8	8	4	8	8	4	4	4	8
	II	4	4	4	8	8	8	4	8	4	4	4	4	4	4
	III	8	4	4	4	8	4	8	4	4	4	4	4	4	n.d.
**Bac7 (1-16)**	I	1	2	2	4	8	16	32	32	64	128	128	128	128	128
	II	1	1	1	4	4	4	8	8	32	32	32	128	256	256
	III	2	4	8	8	16	32	32	32	32	32	64	64	64	128
**Chloramphenicol**	I	16	32	32	32	32	32	64	64	64	64	64	64	128	128
	II	32	8	16	32	32	32	64	64	256	64	64	128	128	128
	III	16	32	64	128	128	512	512	512	512	512	512	512	512	1024
**Colistin**	I	0.5	0.5	0.5	0.5	0.5	0.5	0.5	0.5	0.5	0.5	0.5	0.5	0.5	0.5
	II	0.5	0.5	0.5	0.5	0.5	0.5	0.5	0.5	0.5	0.5	0.5	0.5	0.5	0.5
